# Progression of right ventricular dilation in repaired tetralogy of fallot

**DOI:** 10.1186/1532-429X-15-S1-P281

**Published:** 2013-01-30

**Authors:** Sujatha Buddhe, Amee Shah, Wyman W Lai

**Affiliations:** 1 Pediatric Cardiology, Childrens Hospital of New York, New York, NY, USA

## Background

Right ventricular (RV) dilation shown by cardiac MRI (CMR) is important for determining the timing of pulmonary valve replacement (PVR) in patients with repaired Tetralogy of Fallot (TOF). There is little known about the rate of progression (ROP) of RV dilation and factors that may affect ROP.

## Methods

This is a retrospective cohort study of repaired TOF patients from 01/2004 to 09/2012. All children and young adults with at least two CMR's prior to PVR were included in the study. The ROP of RV dilation prior to PVR was calculated as the difference between the first and last CMR-derived RV indexed end-diastolic volumes (iEDV) divided by the difference in time between the two studies. Subjects were divided into two groups based on the ROP of RV dilation: Group I - subjects with the most rapid ROP (defined as the top quartile of ROP) and Group II - all other subjects (the lower three quartiles of ROP). The differences in characteristic between groups were analyzed.

## Results

A total of 63 subjects with repaired TOF were included in the study, including 30% with a history of pulmonary atresia and 19% with absent pulmonary valve. TOF repair included a transannular patch in 55%, no patch in 16%, and a conduit in 29%. The mean age was 17.9 ± 9.6 years, and mean duration between CMR's was 3.4 ± 2.0 years. Median ROP for RV iEDV was 2.2 (range -14.4 to 35.8) ml/m2/year.

On logistic regression analysis, RV iESV was the only independent predictor of significant ROP of RV dilation. ROC analysis showed an area under the curve of 0.75 (p<0.01). A RV iESV of 54 ml/m2 had 88% sensitivity and 53% specificity in predicting significant ROP, while a cutoff of 67 ml/m2 had 69% sensitivity and 75% specificity.

## Conclusions

There was no significant change in RV iEDV in a majority of repaired TOF subjects. RV iESV was the best predictor of more rapid RV dilation (greater ROP of RV iEDV). Additional studies are needed to better evaluate factors associated with more rapid ROP in repaired TOF.

## Funding

None.

**Table 1:** Group Characteristics:

Variables	Group I (n=16)	Group II (n=47)	p-value
ROP (ml/m2/year) °	13.8 (5.6 to 35.8)	1.2 (-14.5 to 5.4)	<0.01
Age (years) *	17.3 ± 10.3	18.1 ± 9.4	0.8
Male gender Φ	12 (75%)	23 (50%)	0.09
RV iEDV *	153 ± 49	124 ± 31	0.04
RV iESV *	77 ± 29	56 ± 19	0.01
RV EF *	50 ± 7	56 ± 7	0.01
LV iEDV *	74 ± 10	78 ± 15	0.4
PR (moderate to severe) Φ	14 (88%)	39(83%)	0.4
PS (mild+) Φ	3(19%)	14(30%)	0.2
RV outflow tract aneurysm Φ	3 (19%)	13 (28%)	0.7
QRS duration (ms) *	144 ± 28	140 ± 27	0.7
Age at surgery (years) °	0.7 (0.1 to 9.0)	1.5 (0.0 to 7.5)	0.4
Time since surgery (years) *	15.1 ± 8.8	14.5 ± 7.2	0.8
Time between CMR's (years) *	2.2 ± 1.9	3.8 ± 1.9	<0.01

* Mean ± SD; ° Median (range) ; Φ Number (percentage) Abbreviations: EF - ejection fraction, iESV - indexed end-systolic volume, LV - left ventricle, PR - pulmonary regurgitation, PS - pulmonary stenosis, TR - tricuspid regurgitation

